# Effect of *TRAF6*-knockout on gene expression and lncRNA expression in *Epithelioma papulosum cyprini* (EPC) cells

**DOI:** 10.1080/19768354.2023.2263070

**Published:** 2023-10-04

**Authors:** Najib Abdellaoui, Seon Young Kim, Min Sun Kim

**Affiliations:** a Department of Biological Sciences, Kongju National University, Gongju, South Korea; b BK21 Team for Field-oriented BioCore Human Resources Development, Kongju National University, Gongju, South Korea

**Keywords:** *Epithelioma Papulosum Cyprini*, RNA-seq, lncRNAs, immune process

## Abstract

*TRAF6* is a key immune gene that plays a significant role in toll-like receptor signal transduction and activates downstream immune genes involved in antiviral immunity in fish. To explore the role of TRAF6 in *Epithelioma papulosum cyprini* (EPC) cells, we knocked out the *TRAF6* gene using the Clustered Regularly Interspaced Short Palindromic Repeats-Cas9 (CRISPR-Cas9) technique and then analyzed the transcriptomes of the knockout cells. In this study, we identified that 232 transcripts were differentially expressed in naive cells. Using the pipeline, we identified 381 novel lncRNAs in EPC cells, 23 of which were differentially expressed. Gene Ontology enrichment analysis demonstrated that differentially expressed genes (DEG) are implicated in various immune processes, such as neutrophil chemotaxis and mitogen-activated protein kinase binding. In addition, the KEGG pathway analysis revealed enrichment in immune-related pathways (Interleukin-17 signaling pathway, cytokine-cytokine receptor interaction, and TNF signaling pathway). Furthermore, the target genes of the differentially expressed lncRNAs were implicated in the negative regulation of interleukin-6 and tumor necrosis factor production. These results indicate that lncRNAs and protein-coding genes participate in the regulation of immune and metabolic processes in fish.

## 1. Introduction

The innate immune system plays a key role in the detection and recognition of different pathogens and hence in the host’s defense against infection. Pathogen-associated molecular patterns (PAMPs) are recognized by pattern recognition receptors (PRRs), which activate multiple signaling cascades and induce the biosynthesis of downstream effector genes, including Type I interferon (*IFN*) and other cytokines (Akira et al. 2006; Lim and Staudt 2013). Toll-like receptors (TLRs), a family of PRRs that recognize different PAMPs, are classified into different subfamilies. For example, TLR1, TLR2, and TLR6 identify lipids, while, TLR7, TLR8 and TLR9 recognize nucleic acids. Once TLRs are triggered, myeloid differentiation factor 88 (MyD88)-dependent or MyD88-independent pathways are activated, recruiting tumor necrosis factor receptor-associated factor 6 (*TRAF6*) (Rauta et al. 2014; Ahn et al. 2021).

*TRAF6* plays a vital role in innate and adaptive immune responses by controlling downstream signaling pathways depending on the stimuli type. *TRAF6* acts as a signal transducer by activating the transforming growth factor-β-activated kinase 1 (TAK1) binding protein (TAB) complex. Following activation of the TAK1/TAB complex, the NF-κB pathway is triggered, inducing the release of inflammatory cytokines (Takeda and Akira 2004; Liew et al. 2005; Besse et al. 2007). In addition, *TRAF6* regulates the mitogen-activated protein kinase (MAPK) pathway, which participates in cell proliferation, myogenic differentiation, and death (Walsh et al. 2008; Xiao et al. 2012; Wang et al. 2020). *TRAF6* has been characterized in different fish species, including spotted sea bass (Wang et al. 2020), crucian carp (Zhang et al. 2019), rainbow trout (Jang et al. 2019), and zebrafish (Phelan et al. 2005). However, there are more TLRs in fish than in mammals, indicating that fish signaling pathways are more complicated. The fathead minnow (*Pimephales promelas*) is an important fish model that is widely used to study viral infections in aquaculture, as well as the fish immune system. In vitro experiments have demonstrated that *TRAF6* plays an important role in the protection of fathead minnow (FHM) cells against Singapore Grouper Iridovirus infections (Wei et al. 2017). However, the role of TRAF6 in the fathead minnow remains unclear.

In fish, the innate immune system is a vital component of the host’s defense against different pathogens. Therefore, understanding the mechanisms underlying the recognition of pathogens in fish is important for the development of new prophylactic techniques and health management in aquaculture. In this study, we used CRISPR-Cas9 technology to knock out *TRAF6* in EPC cells and compared the gene and long non-coding RNA (lncRNA) expression in *TRAF6*-knockout cells (ΔTRAF6) to that in naïve cells using RNA seq. In addition, we confirmed the transcriptome results by quantitative real-time PCR.

## 2. Materials and methods

### 2.1. Cells

EPC cells (ATCC, No. CRL-2872) were cultured at 25 °C in Lebovitz medium (L-15, Sigma) supplemented with penicillin (100U/ml, Welgene), streptomycin (100 μg/ml, Welgene), and 10% fetal bovine serum (FBS, Sigma).

### 2.2. Vector construction for TRAF6 gene knockout

Using the CRISPR/Cas9 single-guide RNA (sgRNA) design tools (http://www.rgenome.net/cas-offinder/, http://chopchop.rc.fas.harvard.edu/), the best guide RNA (gRNA) sequence targeting the *TRAF6* gene of EPC cells (5′- TGAGTGTCCTATCTGTCTAATGG-3’; PAM, underlined) was designed. To construct the precise ends of the sgRNA expressed by the CMV promoter, the sgRNA scaffold was designed to contain hammerhead ribozymes (HHR) and hepatitis delta virus ribozymes (HdvRz) at the 5’ and 3’ ends. To amplify the sgRNA scaffold containing HHR and HdvRz and the target TRAF6 gene, PCR was first performed using the previously constructed pU6-sgIRF9 vector (Kim et al. 2018) as a template. The primers used for PCR are listed in Table S1. Thermal cycling was initiated with one cycle of 3 min at 95 °C (initial denaturation) followed by 30 cycles of denaturation for 30 s at 95 °C, annealing for 30 s at 60 °C, and extension for 30 s at 72 °C, with a final extension step of 7 min at 72 °C. The amplified fragment was cloned into the pGEM T easy vector (Promega), sequenced, and named RGR-sgTRAF6. RGR-sgTRAF6 was excised by digestion with *Nhe*I and *Xba*I restriction enzymes, ligated into the pcDNA 3.1(+) vector (Invitrogen), and named pc-RGR-sgTRAF6. Using the pc-RGR-sgTRAF6 vector as a template, PCR was performed from the CMV promoter to the BGH polyadenylation signal including the RGR-sgTRAF6 cassette. The PCR product was cloned into the pGEM T easy vector, sequenced, and named CMV-RGR-sgTRAF6-BGHpA. Finally, by digestion with AgeI and EcoRI, the CMV-RGR-sgTRAF6-BGHpA excised fragment was ligated into the Cas9 SmartNucleaseTM all-in-one-tagged vector (System Biosciences) and designated as pCRISPR/Cas9 RGR-sgTRAF6.

### 2.3. Production of TRAF6 gene knockout single-cell clones

To produce *TRAF6* gene knockout cells, pCRISPR/Cas9 RGR-sgTRAF6 was transfected into EPC (3 × 10^6^ cells) using the Neon transfection system (Invitrogen) according to the manufacturer's instructions. Two days post-transfection, the cells were sorted in 96-well plates using an Automated High-speed Cytometry Sorter System (BD FACS Aria III) to obtain single-cell clones. After approximately one month of culture, genomic DNA was isolated from the *TRAF6* knockout cell clones using Quick Extract DNA Extraction Solution (Lucigen) according to the manufacturer's instructions. The fragment containing the 20 bp targeting sequence of the *TRAF6* gene was amplified by PCR using the primer pairs listed in Table S1. PCR products were cloned into the pGEM T Easy Vector and sequenced to detect indels.

### 2.4. RNA-Seq of TRAF6 gene knockout single-cell clones

Total RNA from control cells and the *TRAF6* knockout single-cell clones 35 and 39 was extracted using the Direct-zol RNA Miniprep Kit (Zymo Research) according to the manufacturer's instructions. RNA libraries were prepared and next-generation sequencing was conducted using a DNA link in Korea. The RNA quality and concentration were analyzed using an Agilent 2100 Expert Bioanalyzer. RNA libraries were constructed using a TruSeq Stranded mRNA Prep Kit (Illumina) according to the manufacturer's protocol. The generated libraries were sequenced using NovaSeq 6000 (Illumina) by Macrogen (South Korea).

### 2.5. Next-generation sequencing and data analysis

The generated raw data was analyzed using the OmicsBox tool. Briefly, the quality of raw reads was checked using FastQC, and reads with low quality and adapter sequences were removed using Trimmomatic (Bolger et al. 2014). The clean reads with high quality (QC ≥30), were aligned to the fathead minnow genome (downloaded from the NCBI database) using STAR aligner (Dobin et al. 2013), and the total counting of transcripts was determined using HTSeq (Simon et al. 2014). Counts were normalized and expressed as RPKM (reads per kilobase of transcript per million mapped reads). The expression of levels of various transcripts in ΔTRAF6-cells were compared to those of the naïve cells using the R package NOISeq (Tarazona et al. 2015) and differentially expressed genes (DEG) with *P*-values lower than 0.05 were determined. The protein sequences of fathead minnow were retrieved from the NCBI database and then queried against the NCBI non-redundant (nr) database using the BLASTP tool with a cut-off E-value of ≤10^−5^. The best hits were mapped and annotated, and functional annotations were performed using Blast2GO (Conesa et al. 2005). Gene Ontology and Kyoto Encyclopedia of Genes and Genomes (KEGG) pathway enrichment analyses were performed using the ClusterProfiler package (Wu et al. 2021) and visualization was performed using the ggplot2 package (Wickham 2016).

LncRNAs in fathead minnow were discovered using a previously described pipeline (Chen et al. 2019; Valenzuela-Muñoz et al. 2021). Briefly, first the transcripts with ‘u’ characters were retained as a possible lncRNA using the parameter ‘-r’ in the gffcompare tool (StringTie (Shumate et al. 2022)). Transcripts with a length of less than 200 bp or one exon were eliminated. The protein-coding potential of the remaining transcripts was predicted using CPAT, PLEK, and LncFinder (Wang et al. 2013; Li et al. 2014; Han et al. 2019) and only the intersection of the three tools with the non-coding potential was further analyzed. The sequences of the transcripts were translated using Transeq, and corresponding proteins showing high similarity to those in the Pfam database were removed using pfamscan with an E-value <1e^−5^ (Potter et al. 2018). Finally, any transcript with similarity to a protein in the NCBI, NR, and UniRef90 databases was discarded using BLASTP with an E-value of <1e^−5^ (Boratyn et al. 2013).

### 2.6. Quantitative real-time PCR (qRT-PCR)

To validate the transcriptome expression, five genes were selected and verified by qRT-PCR. Total RNA was extracted from EPC cells and ΔTRAF6-EPC cells using the Direct-zol RNA Miniprep Kit (Zymo Research, USA), following the manufacturer’s instructions. Then, 1 μg of total RNA was reverse transcripted using Reverse Transcription Master mix (ElpisBio, South Korea). The reaction was incubated at 37 °C for 60 min, followed by heat inactivation at 94 °C for 5 min. qPCR was performed on a LightCycler® 96 (Roche Diagnostics, Rotkreuz, Switzerland) with 5 μl of diluted cDNA product, 1μl of forward and reverse primer (Table S1), 10μl of TOPreal™ qPCR 2X PreMIX (Enzynomics, South Korea), and 5μl of DNase-free water. The PCR cycling conditions were as follows: 95 °C for 10 min, 40 cycles of 95 °C for 10 s, 60 °C for 10 s, and 72 °C for 10 s. The relative expression of selected genes was determined using the 2^-ΔΔCT^ method and the beta-actin gene was used as the internal control. Each experiment was performed in triplicate. The primer sequences are listed in Table S1.

## 3. Results

### 3.1. Generation of TRAF6 gene knockout EPC cells

**Figure 1.**
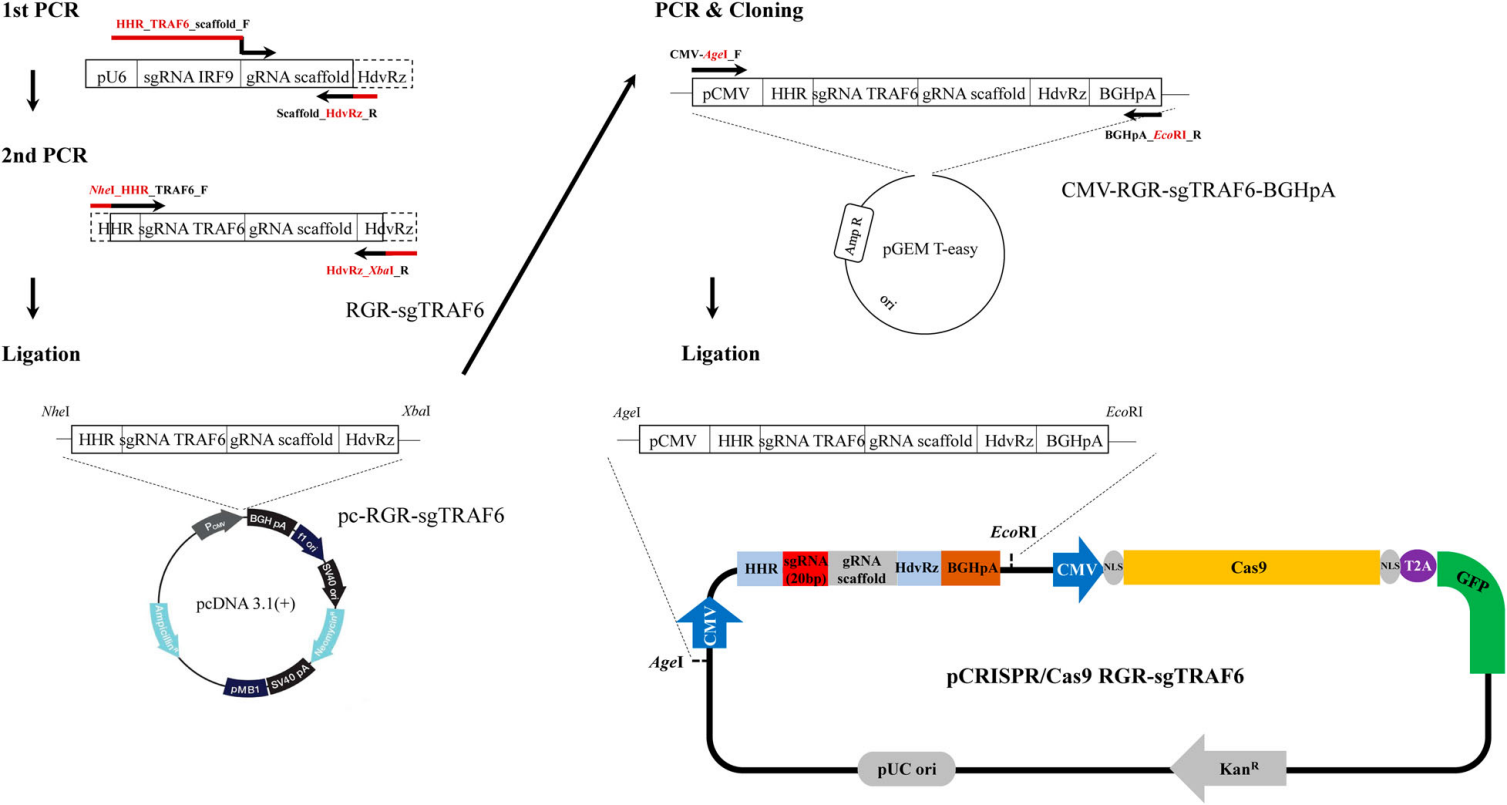
Workflow showing the procedure used to construct the CRISPR/Cas9 vector targeting *Epithelioma papulosum cyprini* (EPC) cell's TRAF6 gene (pCRISPR/Cas9 RGR-sgTRAF6).

**Figure 2.**
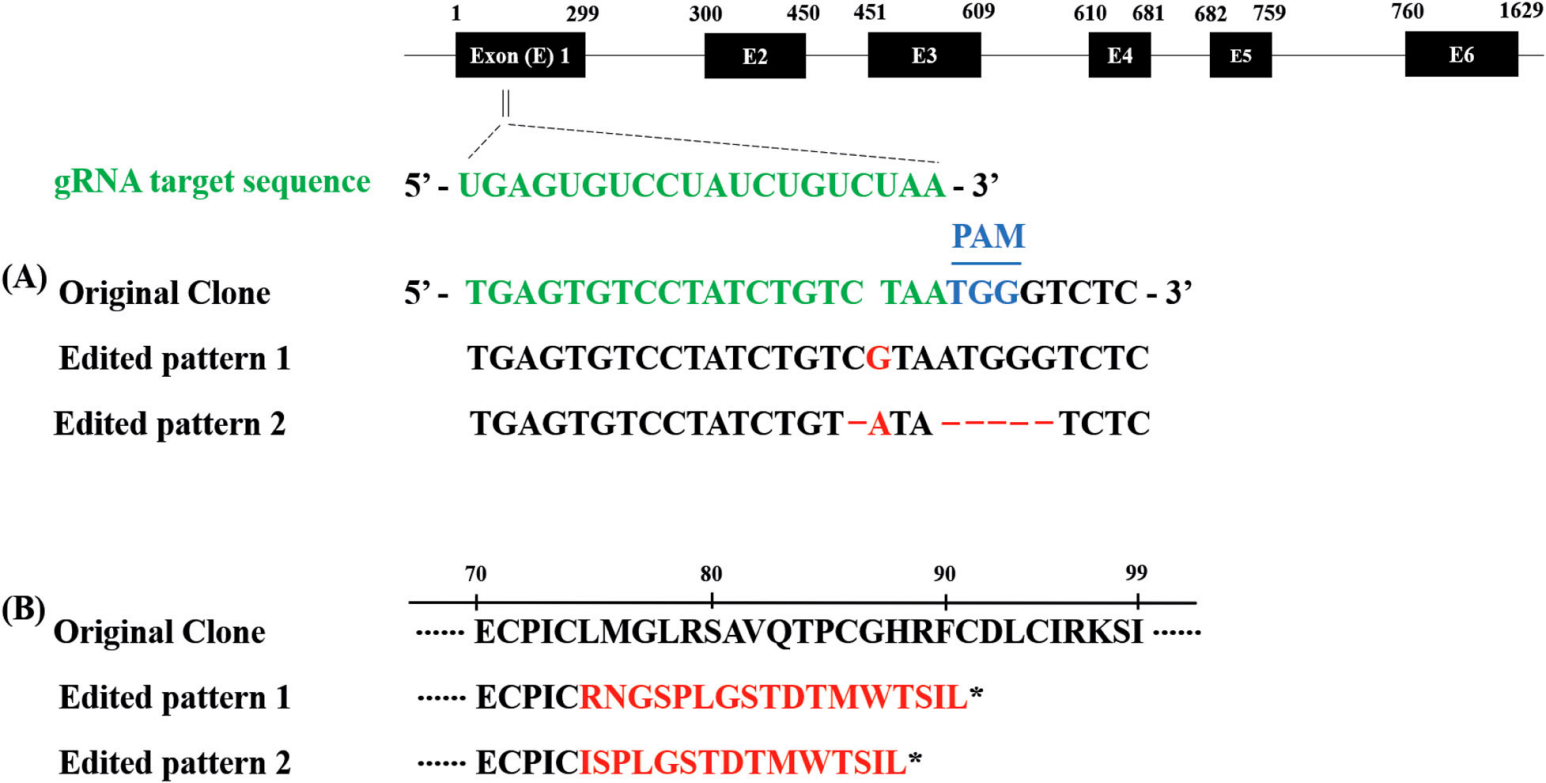
Sequence alignment of nucleotides and amino acids of TRAF6 gene. The knockout of the TRAF6 cell clone showed heterozygous insertion/deletion (indel) mutations. The upper black boxes represent exon regions of the TRAF6 gene and the upper numbers of the boxes mean the nucleotides of TRAF6 ORF from 1 to 1629 (A). The asterisk represents the generation of a premature stop codon by out-of-frame indels (B).

Single-cell clones transfected with the pCRISPR/Cas9_RGR-sgTRAF6 vector (Figure 1) were analyzed by sequencing the target region to confirm the indels. Among the 96 replicates of cell clones with pCRISPR/Cas9 RGR-sgTRAF6, eight clones were cultured, and only two clones showed two heterozygous indels in *TRAF6* (Figure 2A). All heterozygous indels generated premature stop codons (Figure 2B).

### 3.2. RNA-seq profiling of naïve cells and ΔTRAF6-cells

**Figure 3.**
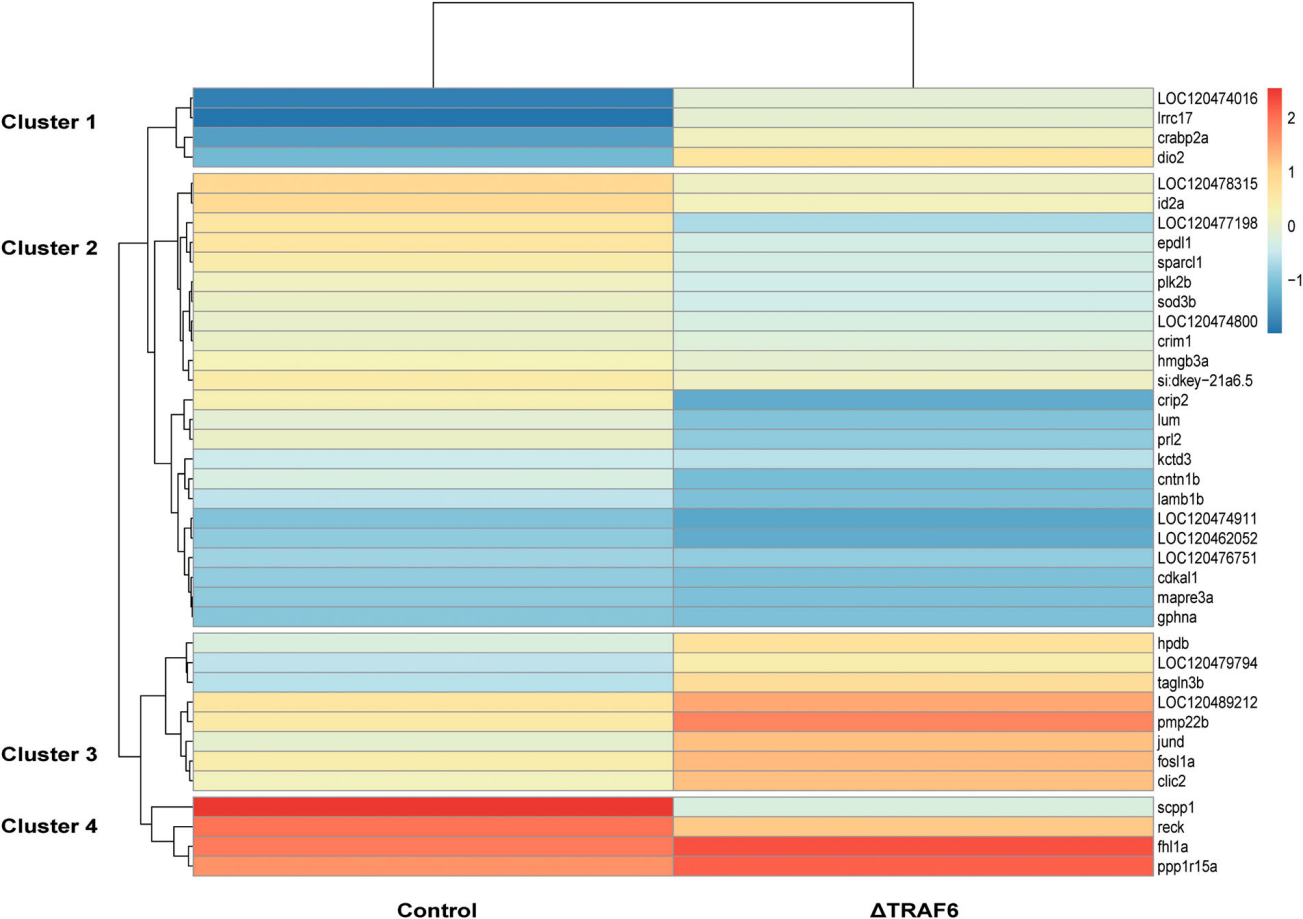
Heatmap of differentially expressed genes in naïve cells (control) and TRAF6-knockout cells(ΔTRAF6). The heatmap was generated using the Complex Heatmap package and the counts of differentially expressed genes. Upregulated genes are indicated in red and downregulated genes are indicated in blue.

Two libraries were constructed to investigate the effects of knockout of *TRAF6* on gene expression in fathead minnow cells (EPC). After removing the adapter sequences and low-quality reads, approximately 35,359,865 and 39,716,592 clean reads were obtained for naïve cells and ΔTRAF6-cells, respectively. Over 92% of the reads were unique, approximately 5% mapped more than once to the genome of fathead minnow, and less than 2.5% were unmapped reads. After alignment with the reference genome, DEGs with *P*-values less than 0.05, and fold changes (FCs) ≥ |1.5| were determined using the R package NOISeq. Accordingly, 232 of the 16,715 annotated transcripts were found to be differentially expressed. In comparison to naïve cells, 58 genes were upregulated and 174 genes were downregulated, and the top 40 genes were visualized using the pheatmap package based on their fold changes (Figure 3). The downregulated genes were more than the upregulated genes, indicating that the knockout of the *TRAF6* gene negatively impacts gene expression. The heatmap showed that the genes could be grouped into four clusters based on their expression (Figure 3). The downregulated genes included Cysteine Rich Protein 2 (*CRIP2*), which is involved in the NF-κB pathway, and Phosphatases of regenerating liver 2 (*PRL2*), which is involved in the regulation of oxidative stress. The upregulated genes *CD74a*, which engages in T cell activation and dendritic cell antigen processing and presentation, and inhbab, which is characterized by cytokine activity, were predicted to be involved in SMAD protein signal transduction (Table S2).

**Figure 4.**
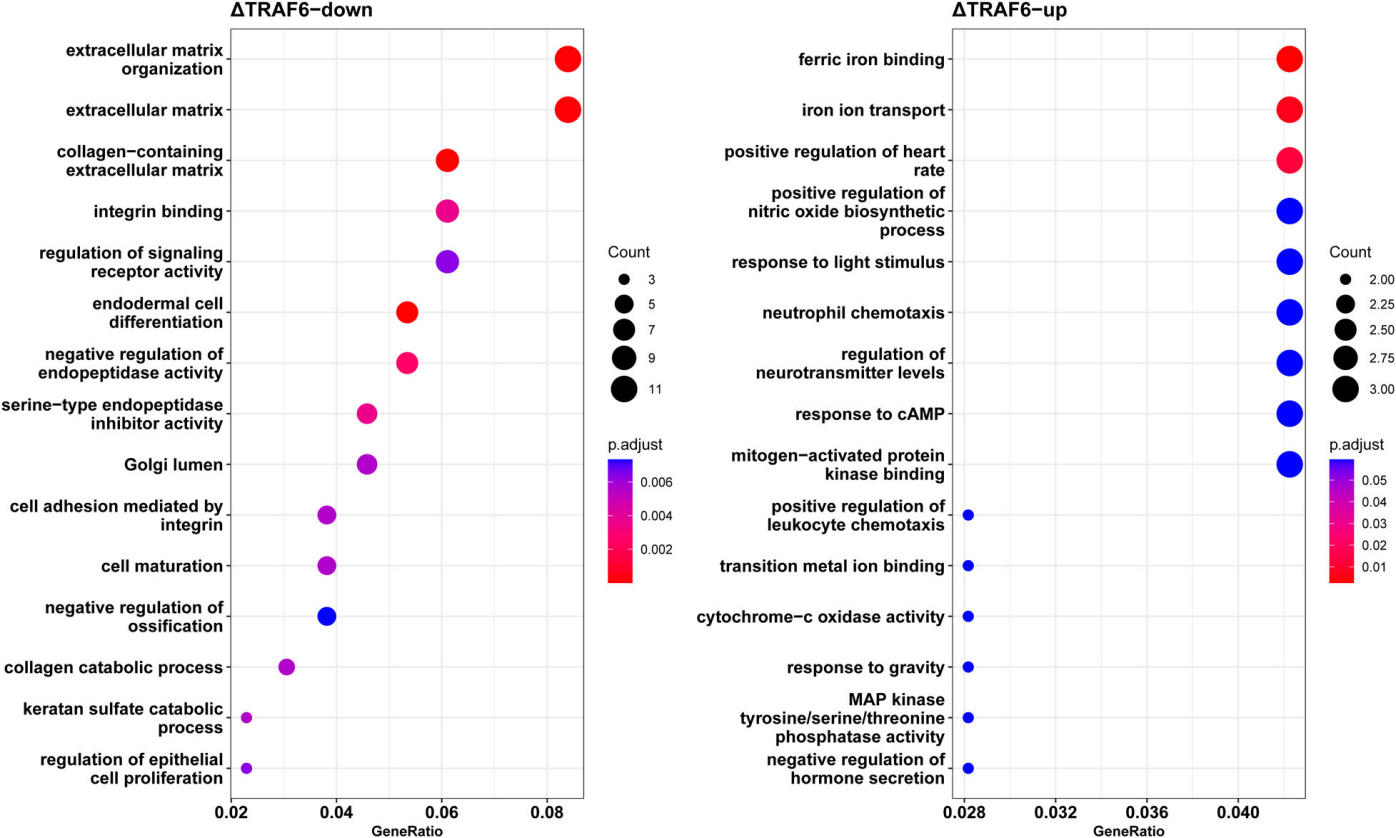
The top 15 enriched gene ontology terms of (A) downregulated genes and (B) upregulated genes. The abscissa represents the proportion to gene ratio of the enriched gene number. The node size represents the count of genes significantly enriched in each gene ontology term.

GO annotation was performed using the BLAST2GO tool against the GO database (http://www.geneontology.org), followed by enrichment and pathway enrichment analyses. GO enrichment analysis showed that 307 GO terms were significantly enriched for downregulated genes and 227 terms were significantly enriched for upregulated genes (Table S3). As shown in Figure 4, the GO terms enriched in downregulated genes were mostly involved in cellular processes, such as extracellular matrix organization, integrin binding, and endodermal cell differentiation (Figure 4A), whereas ferric iron binding, iron ion transport, neutrophil chemotaxis, and mitogen-activated protein kinase binding were enriched in upregulated genes (Figure 4B).

**Figure 5.**
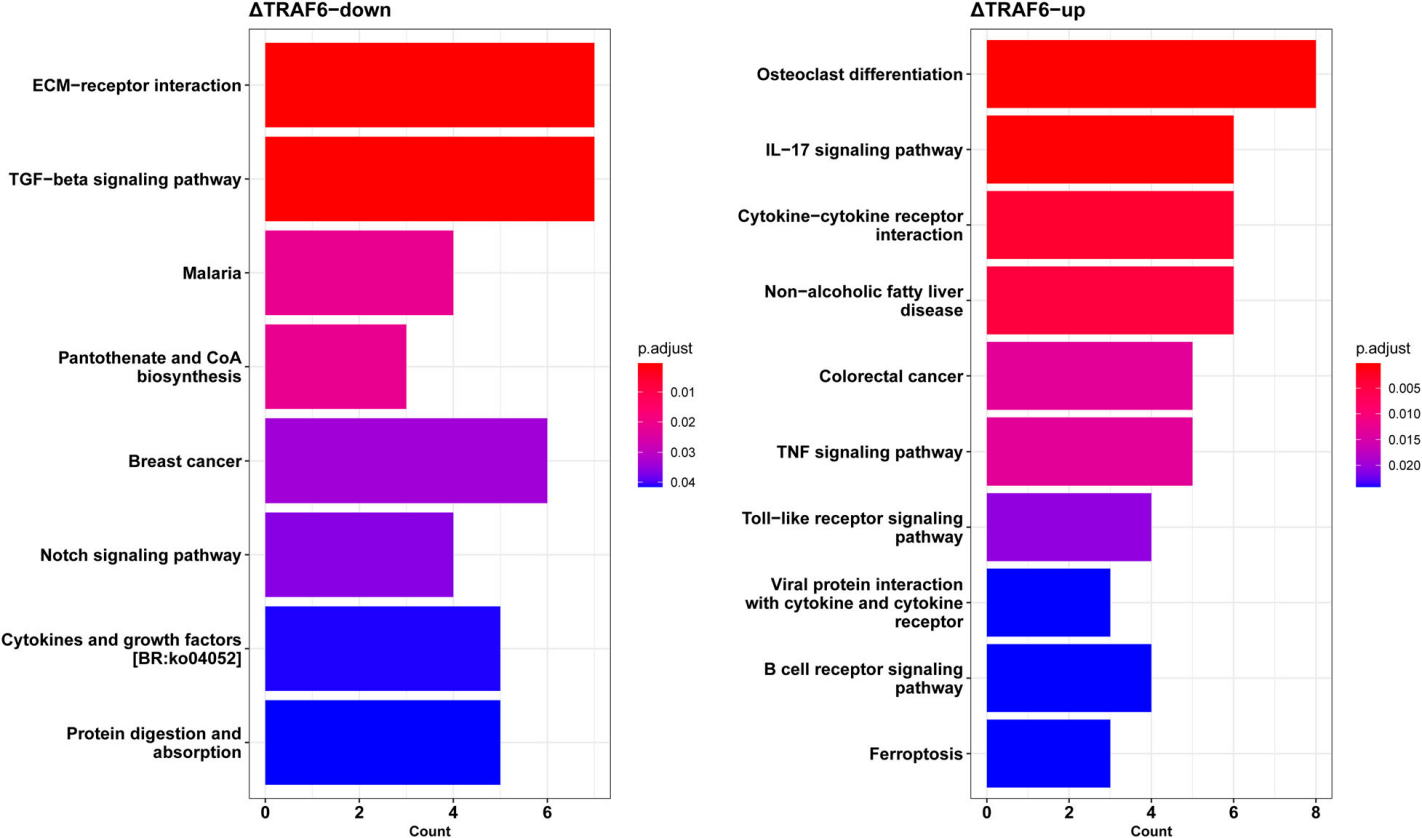
KEGG pathway enrichment analysis of (A) downregulated genes and (B) upregulated genes using ClusterProfiler package. The abscissa represents the count of genes significantly enriched in each pathway.

The KEGG enrichment of DEG identified 27 signaling pathways that were significantly altered in ΔTRAF6-EPC cells. The top 10 KEGG signaling pathways are shown in Figure 5. The downregulated genes were enriched in ECM-receptor interaction, TGF-beta signaling pathway, and malaria (Figure 5A). The upregulated genes were enriched in the IL-17 signaling pathway, cytokine-cytokine receptor interaction, and the TNF signaling pathway (Figure 5B).

### 3.3. Expression profiling of lncRNA in EPC cells

**Figure 6.**
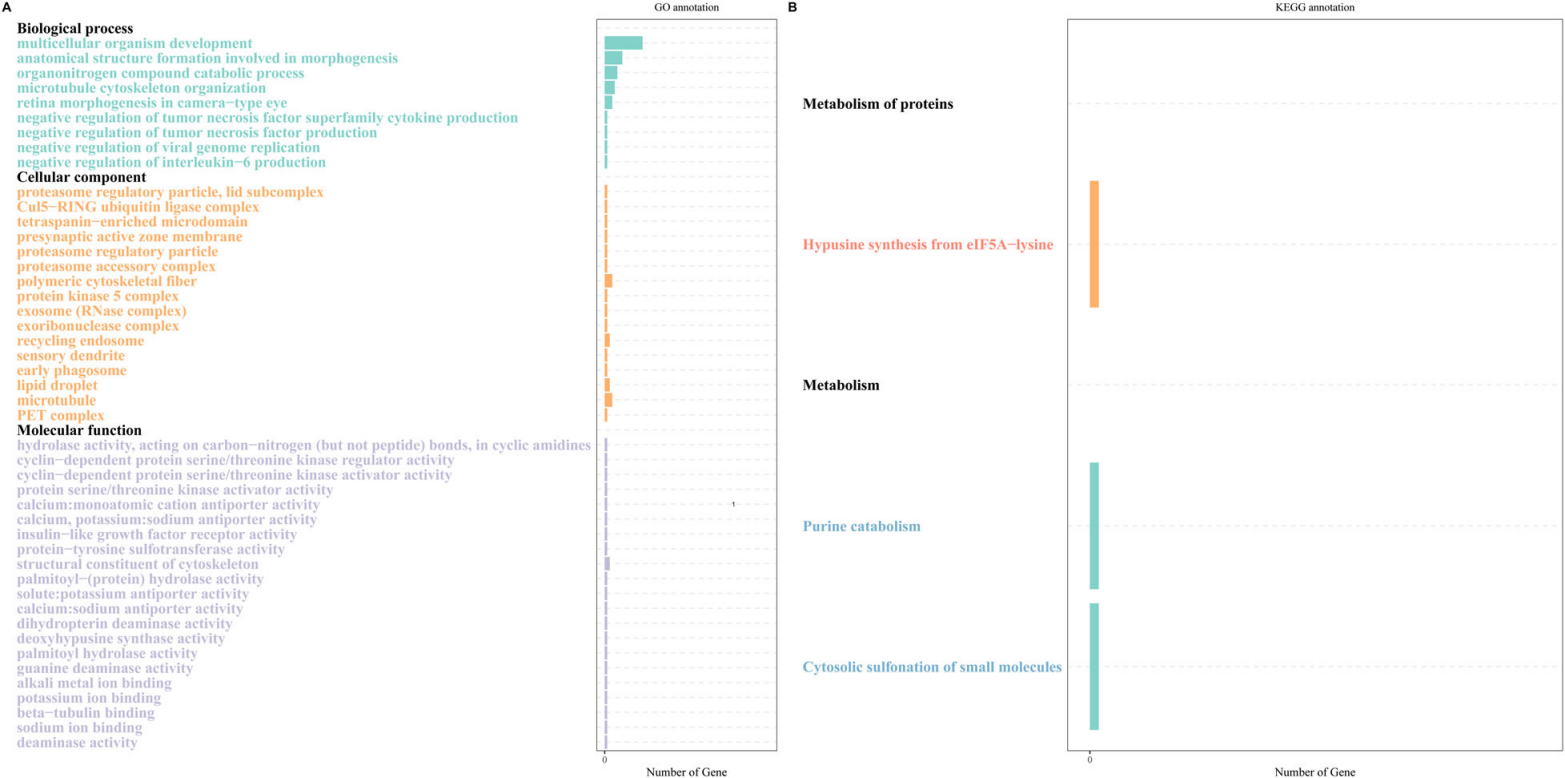
Gene ontology enrichment analysis (A) and KEGG pathway analysis (B) of cis-target genes of differentially expressed lncRNAs. The gene ontology (A) categories were biological process, cellular component, and molecular function.

The first part of our pipeline for the identification of lncRNAs in fathead minnow was the de novo assembly of the alignment files using StringTie and then filtering using Gffcompare. We identified 7,052 novel transcripts. Next, novel transcripts were filtered based on their length (>200 nt) and open reading frame (ORF) evaluation. Their coding potential was estimated using three tools (CPC2, PLEK, and LncFinder) and their intersection, and 1115 novel transcripts were considered in the next step (Fig. S1). Transeq was used to predict the ORF for each novel transcript, and candidate transcripts were blasted against the NR database using BLASTP. Transcripts were discarded when they were denoted as coding sequences using TranSeq and BLASTP. Using this pipeline, we identified 381 novel lncRNA transcripts in fathead minnow. To investigate the effect of TRAF6 knockout on EPC, we analyzed lncRNA expression using the R package NOISeq. Among the 23 lncRNAs that were found to be differentially expressed (Table 1), 10 were upregulated and 13 were downregulated in comparison to the control group with a FC≥|1.5| and a *P*-value less than 0.05. The lncRNA target genes were determined by analyzing the role of Cis lncRNAs on neighboring genes. The coding genes within 10–100 kb upstream or downstream of the lncRNA were considered target genes. Therefore, the cis-target genes of the differentially expressed lncRNAs corresponded to 30 protein-coding genes (Table 1). Among the target genes of lncRNAs, tetraspanin 9a (*tspan9a*) belongs to the tetraspanin family and is involved in signal transduction during cell development, activation, and growth. TAFA chemokine-like family member 5a (*tafa5a*) functions as a chemokine-like protein and stimulates the migration of macrophages through the MAPK3/ERK1 and AKT1 pathways. We performed GO enrichment analysis of the target genes of the differentially expressed lncRNAs to predict their potential functions. Gene ontology enrichment analysis showed that the lncRNAs were associated with various cellular components, such as polymeric cytoskeletal fibers (GO:0099513), microtubules (GO:0005874), and recycling endosomes (GO:0055037). Biological processes were mainly involved in multicellular organism development (GO:0007275), anatomical structure formation was involved in morphogenesis (GO:0048646), and negative regulation of interleukin-6 production (GO:0032715) (Figure 6A). The major categories of molecular functions were structural constituents of the cytoskeleton (GO:0005200), cyclin-dependent protein serine/threonine kinase regulator activity (GO:0016538), and protein serine/threonine kinase activator activity (GO:0043539). KEGG pathway analysis of differentially expressed lncRNAs in ΔTRAF6 cells in comparison to naïve cells revealed three different pathways (hypusine synthesis from eIF5A-lysine, cytosolic sulfonation of small molecules, and urine catabolism) corresponding to the metabolism of proteins pathways (Figure 6B).

**Table 1.** Fold change of differentially expressed lncRNAs and their putative Cis-regulated genes.

	LncRNA ID	Fold change	Chromosome	Target gene	Protein ID
up	MSTRG.15884.4	2.133	NW_024121599.1	tspan9a	XP_039506408.1
up	MSTRG.7520.1	1.732	NW_024121210.1	LOC120482205	XP_039532215.1
up	MSTRG.7520.1	1.732	NW_024121210.1	tpst2	XP_039533351.1
up	MSTRG.7038.4	2.511	NW_024121210.1	LOC120483115	XP_039533940.1
up	MSTRG.9784.1	1.886	NW_024121277.1	tuba8l3	XP_039539361.1
up	MSTRG.9784.1	1.886	NW_024121277.1	LOC120487337	XP_039539583.1
up	MSTRG.9784.1	1.886	NW_024121277.1	mnx2a	XP_039539115.1
up	MSTRG.9784.1	1.886	NW_024121277.1	cfap65	XP_039539066.1
up	MSTRG.20768.1	1.98	NW_024121988.1	LOC120473120	XP_039518730.1
up	MSTRG.20768.1	1.98	NW_024121988.1	dhps	XP_039518698.1
up	MSTRG.2630.7	1.829	NW_024121122.1	sdk2b	XP_039521372.1
down	MSTRG.2177.1	−4.288	NW_024121118.1	srpk2	XP_039520092.1
down	MSTRG.2177.1	−4.288	NW_024121118.1	zgc:114188	-
down	MSTRG.314.2	−1.697	NW_024121099.1	crim1	XP_039523088.1
down	MSTRG.314.2	−1.697	NW_024121099.1	slc24a4a	XP_039523115.1
down	MSTRG.11174.1	−2.252	NW_024121321.1	psmd11a	XP_039543386.1
down	MSTRG.11174.1	−2.252	NW_024121321.1	cdk5r1a	XP_039543717.1
down	MSTRG.1726.1	−4.317	NW_024121108.1	irx1a	XP_039511053.1
down	MSTRG.4740.3	−4.695	NW_024121164.1	LOC120478387	XP_039526179.1
down	MSTRG.19090.1	−2.082	NW_024121876.1	abhd17b	XP_039515241.1
down	MSTRG.19090.1	−2.082	NW_024121876.1	cunh9orf85	XP_039515242.1
down	MSTRG.19090.1	−2.082	NW_024121876.1	gda	XP_039515753.1
down	MSTRG.6434.1	−3.273	NW_024121199.1	stc1	XP_039530706.1
down	MSTRG.1774.1	−4.58	NW_024121110.1	tafa5a	XP_039511327.1
down	MSTRG.16161.2	−2.703	NW_024121621.1	asb2b	XP_039507017.1
down	MSTRG.16161.2	−2.703	NW_024121621.1	LOC120462465	XP_039507048.1
down	MSTRG.16161.2	−2.703	NW_024121621.1	exd1	XP_039507036.1
down	MSTRG.8242.1	−2.426	NW_024121222.1	LOC120484488	XP_039535603.1
down	MSTRG.8242.1	−2.426	NW_024121222.1	LOC120484618	XP_039535833.1
down	MSTRG.8242.1	−2.426	NW_024121222.1	syt11b	XP_039535493.1

### 3.4. Validation of qRT-PCR for differentially expressed genes

**Figure 7.**
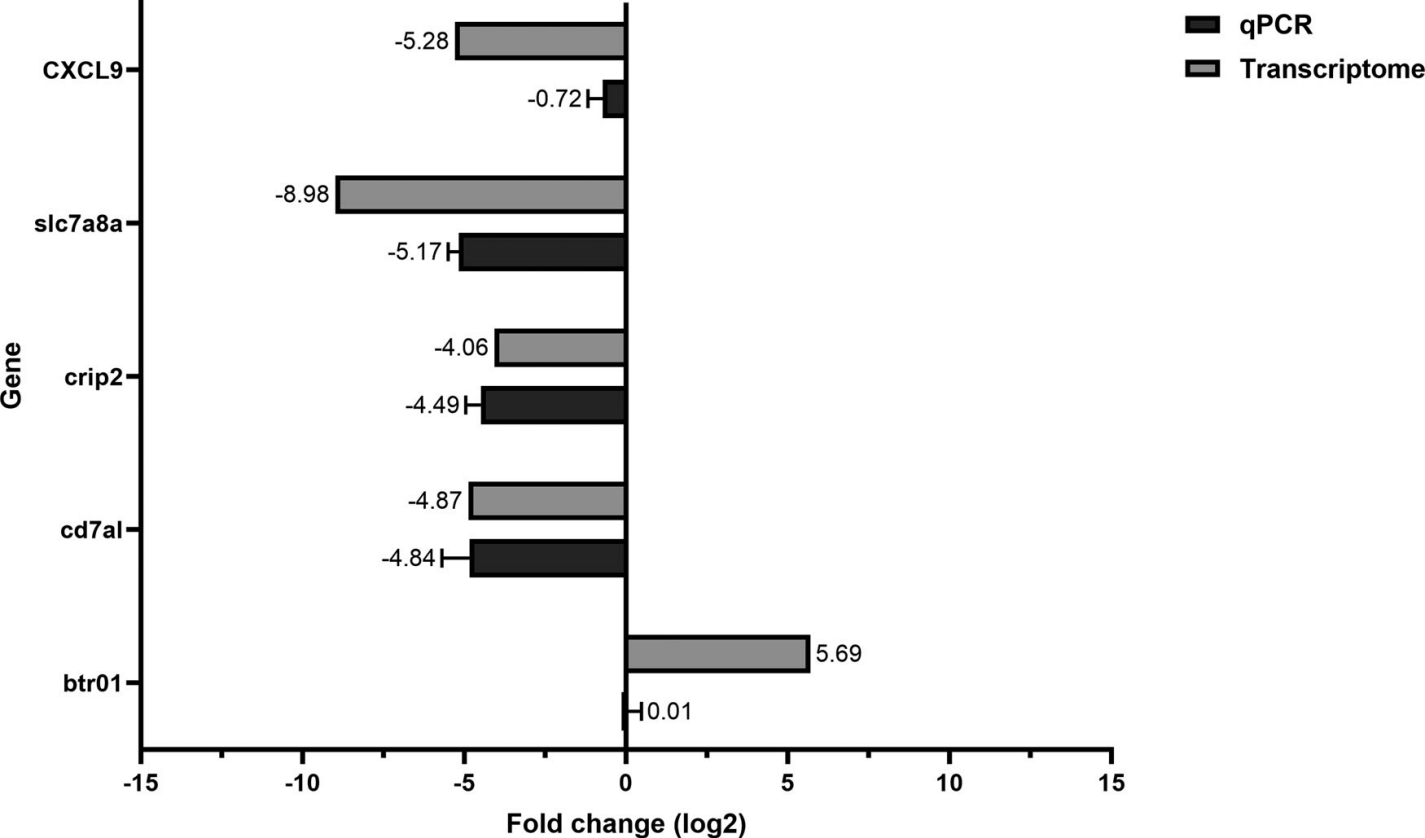
Comparison between transcriptome and qRT-PCR expression levels. The differentially expressed genes were validated by qPCR. The expression level showed a similar pattern to the transcriptome fold change.

To confirm the results of the transcriptome analysis, five upregulated and downregulated genes were selected. The qRT-PCR results showed that the five genes had similar expression levels in comparison with the fold change of RNA-seq (Figure 7).

## 4. Discussion

The TNF receptor-associated factor 6 gene belongs to the TRAF family which activates NF-kappa-B through the activation of the IKK complex. At the same time, *TRAF6* plays a role in signal transduction via the TNF and IL-1 receptors (Wei et al. 2017; Liyanage et al. 2020; Wang et al. 2020). However, its activity is coordinated through the interactions of its regulatory genes with other genes. For example, the lncRNA LTCONS5539 modulates innate immunity through the upregulation of *TRAF6* in the mouse croaker (Pan et al. 2022). In addition, Gao et al. (2021) demonstrated that miR-2187 modulates the NF-κB and IRF3 pathways by targeting *TRAF6* in fish. Therefore, in the present study, we knocked the *TRAF6* gene through the use of CRISPR-cas9 technology. Subsequently, we conducted transcriptome analysis of mRNA and lncRNA expression profiles in *TRAF6* knockout cells and naïve EPC cells to investigate the role of *TRAF6* and its role in immunity. In this study, the knockout of *TRAF6* deregulated gene expression, resulting in 232 differentially expressed genes. Among the DEG, 58 genes were upregulated and 174 genes were downregulated. The higher number of downregulated genes indicates that TRAF6 KO negatively impacts gene expression. Among the DEG, Chemokine (C-X-C motif) ligand 9 (*CXCL9*) and C–C motif chemokine 20b (*ccl20b*) genes were downregulated with a fold change of −5.3 and –3.5, respectively. The expression of *CXCL9,* an important chemokine involved in innate immune responses (van der Aa et al. 2012; Boison et al. 2019), is induced by IFN-gamma which stimulates the NF-κb signaling pathway. In addition, Miki et al. reported that *STAT1* and *NF-κB* can cooperate and regulate the expression of *CXCL9* (Hiroi and Ohmori 2003; Ding et al. 2016). The chemokine *ccl20b* plays an important role in viral and bacterial infections in fish (Cao et al. 2019) and is upregulated via *STAT6* (Ran et al. 2018) which is stabilized by binding to *TRAF6*, reducing its ubiquitination (Zhou et al. 2020). These results indicate that the knockout of the *TRAF6* gene could downregulate chemokine gene expression. Among the upregulated genes, the cAMP-responsive element modulator gene showed upregulated expression, with a fold change of 2.9. The cAMP-responsive element modulator gene (*CREM*) plays a critical role in T lymphocytes through its interaction with gene promoters, regulating the production of chemokines via the NF-κB signaling pathway (Wen et al. 2010; Rauen et al. 2013). In addition, *CREM* is involved in the regulation of T cells (Subramanyam and Tenbrock 2021). In addition, caveolin-1, dual-specificity protein phosphatase 8, and dual-specificity protein phosphatase 2 were upregulated following *TRAF6* knockout. These genes have been implicated in the regulation of the innate and adaptive immune systems in fish (Guo et al. 2011; Li et al. 2017; Lang and Raffi 2019) through negative feedback on MAPK signaling. The knockout of TRAF6 inhibits the activation of mitogen- and stress-activated protein kinase (MSK) genes, activating the dual-specificity protein phosphatases (Ananieva et al. 2008; Reyskens and Arthur 2016; Ding et al. 2018).

To understand the interactions of differentially expressed genes, an enrichment analysis of the GO and KEGG pathways was performed. GO enrichment analysis showed that several biological terms were enriched, including immune pathways such as neutrophil chemotaxis, mitogen-activated protein kinase binding, and positive regulation of leukocyte chemotaxis. *TRAF6* is involved in the posttranslational regulation of regulatory T cells through the transcription of FOXP3 (Ni et al. 2019). In addition, KEGG pathways were mainly enriched in immune pathways such as the IL-17 signaling pathway, cytokine-cytokine receptor interaction, TNF signaling pathway, and Toll-like receptor signaling pathway.

In addition to protein-coding genes, long-noncoding RNA (lncRNAs) are RNA molecules with a length of over 200 nt that possess various functions in regulating gene transcription, epigenetic regulation, and cell proliferation and differentiation (Esteller 2011; Chen et al. 2022). Recently, several studies have investigated the roles of lncRNAs in the immunity of fish (Wang et al. 2018; Leiva et al. 2020; Zheng et al. 2021a; Haridevamuthu et al. 2023). Zheng et al. (2022) demonstrated that the lncRNA MIR122HG inhibits the innate immune response by negatively regulating the TAK1 pathway. Furthermore, the lncRNA NARL plays a critical role in the regulation of the innate immune response to bacterial and viral infections (Zheng et al. 2021b). Boltaña et al. (2016) explored the effect of viral infection with infectious salmon anemia virus on lncRNA expression in the gill, head kidney, and liver and reported that 3,294, 3,275, and 3,325 lncRNAs, respectively, were differentially expressed in different organs. In this study, 381 novel lncRNAs were identified, 23 of which were differentially expressed. In general, lncRNA sequence motifs lack the information required to predict their function (Zhang et al. 2016). Therefore, lncRNA function was analyzed in this study through their cis-action on neighboring protein-coding genes. GO analysis of cis-target genes of differentially expressed lncRNAs revealed enrichment of immune-related terms, such as regulation of phagosome maturation, negative regulation of interleukin-6 production, and negative regulation of tumor necrosis factor production, which have been reported to be regulated by the *TRAF6* gene (Nagashima et al. 2018). Pan et al. (2022) investigated the role of lncRNA LTCON5539 and demonstrated that LTCON5539 increased the expression of antiviral genes and chemokines via the upregulation of *TRAF6* gene expression. Additionally, IRL stimulates the NF-κB signaling pathway by binding to miR-27c-3p in fish (Zheng et al. 2021a). Therefore, differentially expressed lncRNAs may be involved in the regulation of immunity in fish. In this study, the KEGG pathway analysis of the target genes of the differentially expressed lncRNAs was enriched in the metabolism and metabolism of protein pathways. This confirmed that lncRNAs have diverse functions in the regulation of immunity and metabolism in fish. It has been reported that lncRNAs are associated with lipid metabolism in tilapia and the marine Teleost *Cynoglossus semilaevis* (Tao et al. 2022).

In this study, we knocked out the TRAF6 in EPC cells to investigate its role in innate immunity and to study the signal transducer role of TRAF6 in different signaling pathways (NF-κB pathway, MyD88 pathway, IL-17 signaling pathway, and Toll-like receptor signaling pathway). The results showed that knockout induced the downregulation of several immune genes such as chemokine (C-X-C motif) ligand 9 (*CXCL9*) and C–C motif chemokine 20b (*ccl20b*). Therefore, we can conclude that the knockout of *TRAF6* in EPC induces strong modulation of protein-coding genes, as well as modulation of lncRNAs that have a potential role in immune regulation. Furthermore, the investigation of lncRNA function in fish could help in understanding the underlying mechanisms of their immune response to viral and bacterial infections.
